# Cytoreductive surgery and HIPEC for multicystic peritoneal mesothelioma: therapeutic approach in a rare benign neoplasm

**DOI:** 10.1093/jscr/rjaf1041

**Published:** 2026-01-08

**Authors:** Goran Aleksandrić, Nemanja Trifunović, Jovana Trifunović, Sara Filipović, Nebojša Mitrović

**Affiliations:** Department of General Surgery, Clinical Hospital Center Zemun, Vukova 9, Zemun, Belgrade 11000, Serbia; Faculty of Medicine, University of Belgrade, Dr Subotića 8, Savski Venac, Belgrade 11000, Serbia; Department of General Surgery, Clinical Hospital Center Zemun, Vukova 9, Zemun, Belgrade 11000, Serbia; Clinical Hospital Center Zemun, Oncology Hospital, Vukova 9, Zemun, Belgrade 11000, Serbia; Clinical Hospital Center Zemun, Oncology Hospital, Vukova 9, Zemun, Belgrade 11000, Serbia; Department of General Surgery, Clinical Hospital Center Zemun, Vukova 9, Zemun, Belgrade 11000, Serbia; Faculty of Medicine, University of Belgrade, Dr Subotića 8, Savski Venac, Belgrade 11000, Serbia

**Keywords:** multicystic peritoneal mesothelioma, cytoreductive surgery, HIPEC, peritonectomy, mesothelial tumor, case report

## Abstract

Multicystic peritoneal mesothelioma (MCPM) is an uncommon tumor of mesothelial origin, characterized by multiple interconnected cystic spaces and uncertain biological behavior. Although traditionally considered benign, its tendency to recur and occasionally spread within the peritoneal cavity challenges this perception. We describe a 32-year-old woman who experienced gradually worsening pelvic pain and a constant feeling of pressure, which intensified after childbirth. Imaging revealed a large multilocular cystic mass occupying the pelvis. At laparotomy, a gelatinous, multilocular lesion was found adherent to the pelvic peritoneum, uterus, ovaries, and rectosigmoid colon. Complete cytoreductive surgery was performed, including pelvic peritonectomy, omentectomy, appendectomy, and resection of the affected serosa, followed by closed-abdomen hyperthermic intraperitoneal chemotherapy (HIPEC) with oxaliplatin (300 mg/m^2^, 42°C, 90 min). Histopathology confirmed benign multicystic mesothelioma with a mesothelial immunophenotype. The patient recovered well and remains disease-free at 6 months. MCPM requires complete cytoreduction, and HIPEC may help achieve lasting disease control.

## Introduction

Multicystic peritoneal mesothelioma (MCPM) is a rare tumor arising from the peritoneal mesothelium. It usually follows a borderline or indolent course. Mennemeyer and Smith first described it in 1979, noting its multilocular cystic pattern and distinction from lymphangioma [1, 2]. Even today, MCPM accounts for ˂1 % of all peritoneal mesotheliomas, with only a few hundred cases reported worldwide.

The majority of reported cases occur in women of reproductive age, supporting a possible hormonal contribution. Proposed mechanisms include estrogen-driven mesothelial proliferation and chronic inflammation [3, 4]. However, growing evidence supports a truly neoplastic origin. Recurrence and even de novo disease have been described in patients without previous surgery or trauma [5, 6]. The presence of estrogen and progesterone receptors, and occasional response to tamoxifen, further support this link [7].

Clinically, MCPM may cause vague abdominal pain, distension, or a slowly enlarging pelvic mass, and is often discovered incidentally [5]. Imaging typically shows a thin-walled multilocular cystic lesion, which can resemble lymphangioma, pseudomyxoma peritonei, or ovarian cystadenoma [8]. Histology confirms mesothelial origin through calretinin, WT1, and cytokeratin 5/6 positivity [5].

Although sometimes regarded as benign, recurrence after partial excision is frequent, occurring in up to half of cases [9]. The introduction of cytoreductive surgery (CRS) with hyperthermic intraperitoneal chemotherapy (HIPEC) has markedly improved long-term outcomes [2, 3, 6]. Current PSOGI/EURACAN guidelines classify MCPM as a borderline peritoneal tumor and recommend complete cytoreduction with or without HIPEC [6, 10].

This case highlights durable disease control achieved with CRS and HIPEC and the value of multidisciplinary care in specialized centers.

## Case report

A 32-year-old woman came to the Department of General Surgery of our institution, because of several months of intermittent lower abdominal and pelvic pain. She also complained of a constant sensation of pelvic pressure that became especially uncomfortable during bowel movements. The symptoms had started during pregnancy and gradually worsened after childbirth. She had no previous abdominal surgery, significant comorbidities, or other medical problems. Routine laboratory results, including tumor markers, were within normal limits.

**Figure 1:**
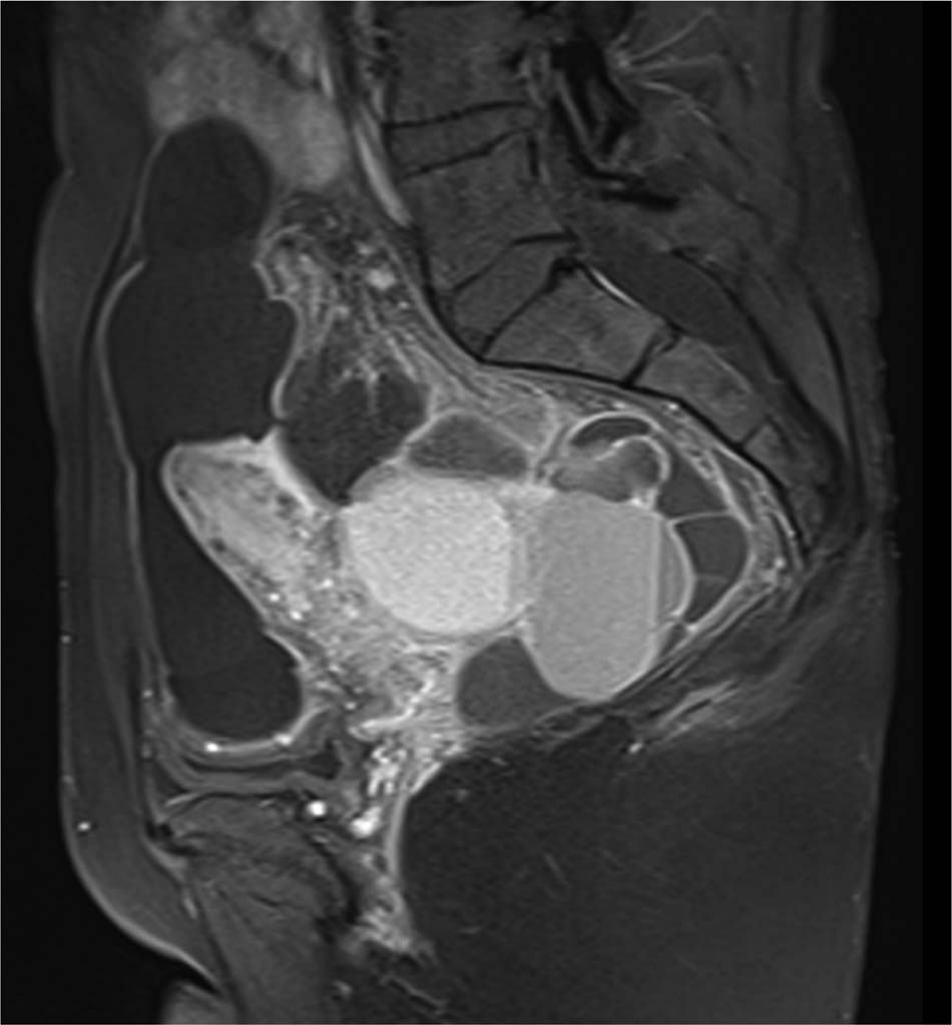
Sagittal T2-weighted pelvic MRI demonstrating a large multilocular cystic lesion occupying the pelvic cavity and displacing adjacent organs.

**Figure 2:**
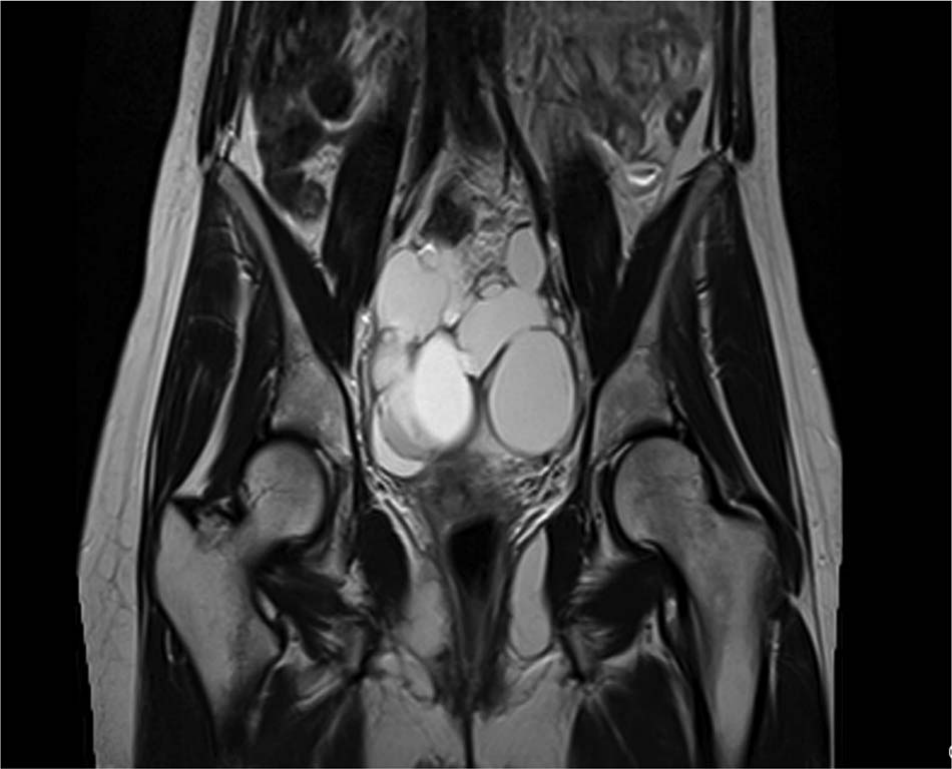
Coronal T2-weighted pelvic MRI showing a large multilocular cystic lesion occupying the pelvic cavity and displacing adjacent organs.

Pelvic ultrasound showed a large multicystic lesion occupying most of the pelvic cavity and pressing on nearby organs. Magnetic resonance imaging (MRI) provided a clearer picture, revealing a multilocular cystic mass displacing the uterus, ovaries, and rectosigmoid colon (Figs 1 and 2). Some cystic areas contained clear fluid, while others showed haemorrhagic or proteinaceous content, raising suspicion of a mucinous cystadenoma or borderline ovarian tumor.

Exploratory laparotomy through a Pfannenstiel incision exposed a gelatinous multilocular mass densely adherent to the pelvic peritoneum, uterus, ovaries, appendix, and rectosigmoid colon. The lesion was carefully removed in one piece, and biopsies were taken from the peritoneum and both ovaries for histological evaluation. Because the disease involved several peritoneal surfaces, complete CRS was carried out, including pelvic peritonectomy, omentectomy, appendectomy, and resection of affected serosa. This was followed by HIPEC with oxaliplatin (300 mg/m^2^) at 42°C for 90 min using the closed-abdomen technique.

Cytology of the cystic fluid showed reactive mesothelial cells without malignancy. Histology revealed cystic spaces lined by flattened to cuboidal mesothelial cells without atypia or mitoses, supported by fibrous stroma. Immunohistochemistry confirmed mesothelial differentiation, with strong CK7, WT1, calretinin, and D2-40 expression, and negative CEA, Ber-EP4, and EMA.

Recovery was smooth. The patient regained bowel function early, resumed oral intake on the second day, and was discharged home a week after surgery. Follow-up MRI scans at three and 6 months showed no recurrence. She remains well and continues regular follow-up.

## Discussion

MCPM remains a rare and intriguing condition. Its slow but persistent behavior makes it one of the more enigmatic entities encountered in peritoneal surgery. Because of its rarity, there are still no standardized guidelines, and most evidence comes from single-center experiences [11].

In the past, MCPM was treated by simple excision or limited debulking, which provided only temporary relief, with recurrence rates approaching 50% [11]. Over time, the strategy evolved into a more comprehensive approach. Today, CRS combined with HIPEC is regarded as the treatment of choice, aiming to achieve both macroscopic clearance and local eradication of residual disease.

Long-term results from specialized centers have been highly encouraging. Zahid *et al*. reported a median recurrence-free survival of over 9 years in 40 patients treated with CRS and HIPEC, without any procedure-related deaths [2]. Similar findings were observed by Nizri *et al*. and Padmanabhan *et al*., who demonstrated a reduction in recurrence rates to ~20% when HIPEC was added to surgery [3, 9]. A multicentre study by Kepenekian *et al*. confirmed this benefit, showing relapse rates of 14% versus 32% after CRS alone [6].

These outcomes align with PSOGI/EURACAN recommendations [10], which classify MCPM as a borderline peritoneal tumor and advocate complete cytoreduction—with or without HIPEC—in high-volume centers.

Careful follow-up remains essential, as late recurrences and rare malignant transformation may occur. Biological factors also seem to influence the disease, including chronic inflammation and hormonal activity in women of reproductive age [11, 12].

Although HIPEC may appear excessive for a histologically benign lesion, published data consistently support its safety, with very low morbidity and no treatment-related mortality when performed by experienced teams [2, 9]. Taken together, CRS combined with HIPEC currently represents the most effective and durable treatment option for extensive or recurrent MCPM, supported by contemporary evidence and international consensus [2, 6, 10, 13].

## Conclusion

MCPM should not be considered entirely benign. Complete CRS remains the key to achieving long-term control, while the addition of HIPEC can further reduce recurrence risk in selected cases. Owing to its slow but persistent nature, careful long-term follow-up is crucial to ensure early detection and maintain favorable outcomes.
